# Synergic Effect of Novel WS_2_ Carriers Holding Spherical Cobalt Ferrite @cubic Fe_3_O_4_ (WS_2_/s-CoFe_2_O_4_@c-Fe_3_O_4_) Nanocomposites in Magnetic Resonance Imaging and Photothermal Therapy for Ocular Treatments and Investigation of Corneal Endothelial Cell Migration

**DOI:** 10.3390/nano10122555

**Published:** 2020-12-19

**Authors:** Shadie Hatamie, Po-Jen Shih, Bo-Wei Chen, I-Jong Wang, Tai-Horng Young, Da-Jeng Yao

**Affiliations:** 1 College of Medicine, National Taiwan University, Taipei 10048, Taiwan; ijong@ntu.edu.tw; 2 Department of Biomedical Engineering, National Taiwan University, Taipei 10617, Taiwan; thyoung@ntu.edu.tw; 3 Institute of Nanoengineering and Microsystem, National Tsing Hua University, Hsinchu 30013, Taiwan; s106035802@m106.nthu.edu.tw (B.-W.C.); djyao@mx.nthu.edu.tw (D.-J.Y.)

**Keywords:** WS_2_/s-CoFe_2_O_4_@c-Fe_3_O_4_ nanocomposites, MRI, BCECs, photo thermal therapy

## Abstract

The design of novel materials to use simultaneously in an ocular system for driven therapeutics and wound healing is still challenging. Here, we produced nanocomposites of tungsten disulfide carriers with spherical cobalt ferrite nanoparticles (NPs) as core inside a cubic iron oxide NPs shell (WS_2_/s-CoFe_2_O_4_@c-Fe_3_O_4_). Transmission electron microscopy (TEM) confirmed that 10 nm s-CoFe_2_O_4_@c-Fe_3_O_4_ NPs were attached on the WS_2_ sheet surfaces. The cytotoxicity of the WS_2_ sheets and nanocomposites were evaluated on bovine cornea endothelial cells (BCECs) using 3-(4,5-dimethylthiazol-2-yl)-2,5-diphenyltetrazolium bromide (MTT) assay for a duration of three days. The MTT assay results showed low toxicity of the WS_2_ sheets on BCECs by 67% cell viability at 100 μg/mL in 24 h, while the nanocomposites show 50% cell viability in the same conditions. The magnetic resonance imaging (MRI) of nanocomposites revealed the excellent T_2_-weighted imaging with an r_2_ contrast of 108 mM^−1^ S^−1^. The in vitro photothermal therapy based on WS_2_ sheets and WS_2_/s-CoFe_2_O_4_ @c-Fe_3_O_4_ nanocomposites using 808 nm laser showed that the maximum thermal energy dispatched in medium at different applied power densities (1200 mw, 1800, 2200, 2600 mW) was for 0.1 mg/mL of the sample solution. The migration assay of BCECs showed that the wound healing was approximately 20% slower for the cell exposed by nanocomposites compared with the control (no exposed BCECs). We believe that WS_2_/s-CoFe_2_O_4_@c-Fe_3_O_4_ nanocomposites have a synergic effect as photothermal therapy agents for eye diseases and could be a target in an ocular system using MRI.

## 1. Introduction

The new generation of two dimensional (2D) materials called transition metal dichalcogenide (TMDC) consists of hexagonal layers of metal atoms between two layers of the chalcogen atoms which have the ability to observed microwave and near infrared (NIR) wave lengths [1,2,3]. These novel two-dimensional sheets such as MoS_2_, MoSe_2_, WS_2_, and WSe_2_, exhibit large surface area and show a boundary effect, resulting in notable electronics and photonics properties [4,5,6]. In addition, the TMDC semiconductors have direct band gaps in the visible and infrared regimes with giant light matter coupling properties and thus become a suitable candidate for the optoelectronics and optics applications [7,8,9]. Among the TMDCs, tungsten disulfide (WS_2_) sheets warrant particular attention because of their non-toxicity, high thermal degradation and high resistance against oxidations [10,11]. The WS_2_ sheets have high NIR absorption capability to use for photothermal treatments in cancer therapy [9,12]. The laser-assisted therapeutics for in vivo and in vitro treatments can be more effective while coupled with materials with high NIR absorbance [13,14,15,16]. In this regard, various types of nanoparticles (NPs) are found to assist laser treatments specially for treatment of eye diseases [17,18]. Sauvage et al. reported on applying laser to ablate the plasmonic NPs conjugated with the vitreous opacities as feasible treatments for eye diseases [19]. In other research, laser has been used to trigger graphene nanosheets to release the drugs inside the eyes after administrations [20,21,22]. To navigate therapeutics, in NPs movements inside the eyes, the use of non-invasive medical diagnostic tools like magnetic resonance imaging (MRI) is promising. To target the nanodevices by MRI, the novel design of nanocomposites for theragnostic applications in biomedicine is of interest. Hatamie et al. designed graphene (2D)/cobalt nano composites for both cancer therapy and MRI imaging [23], while Shahsavari Alavijeh et al. synthesized the molybdenum disulfide/cobalt ferrite (MoS_2_/CoFe_2_O_4_) nanocomposites to use as contrast agents for MRI in cancer treatments [24]. To induce the magnetic properties into the 2D carriers (graphene, TMDCs), magnetic nanoparticles (MNPs) with better magnetic properties are required. Besides, iron oxide MNPs (Fe_3_O_4_) in magnetite phase have great potential in therapeutic and advanced diagnostics because of their high saturation magnetization [25,26,27]. Also, the spinel ferrimagnetic cobalt ferrite MNPs (CoFe_2_O_4_) have high curie temperature (~400 °C), high magnetic anisotropy, and high coercivity compared to those of magnetite NPs [28,29,30]. Interestingly, the combination of MNPs named bi-magnetic core-shell NPs are used in multiferroic technologies and MRI bioimaging because of their unique properties such as high exchange bias, tunable coercivity, blocking temperatures, and large resonance fields compared to the single MNPs [31,32]. The magnetic properties of the core-shell nanostructures are reported to improve due to the diverse coupling interactions between the core and shell nano particles via interfacial defect acts as anisotropy in ferro-ferri and ferro-anti ferro magnetic structures [33,34,35], which make them great candidates to use in magnetic imaging [36,37,38]. In this study, we used the seed-growth technique to prepare spherical cobalt ferrite (s-CoFe_2_O_4_) as a magnetic core template to cover with the cubic iron oxide as shell (c-Fe_3_O_4_) to form (s-CoFe_2_O_4_@c-Fe_3_O_4_) core-shell NPs [39,40,41,42]. The WS_2_ nanosheets were synthesized by a series of chemical methods followed by a non-oxygen annealing process. Furthermore, the nanocomposites of the WS_2_/s-CoFe_2_O_4_@c-Fe_3_O_4_ are prepared with the facile bath sonication of as-synthesized materials. The morphology of the WS_2_, core-shell NPs, and nanocomposites have been characterized using TEM. The cytotoxicity of the WS_2_ and nanocomposites are evaluated using MTT assay on BCECs. The cell migration assay for wound healing was done on the BCECs exposed with nanocomposites. The in vitro photothermal measurements were done on nanocomposites and WS_2_ for comparison. These WS_2_/s-CoFe_2_O_4_@c-Fe_3_O_4_ nanocomposites have great potential for use in theragnostic applications as therapeutics and bioimaging material [43].

## 2. Result and Discussion

### 2.1. TEM of WS_2_/s-CoFe_2_O_4_@c-Fe_3_O_4_ Nanocomposites 

**Figure 1:**
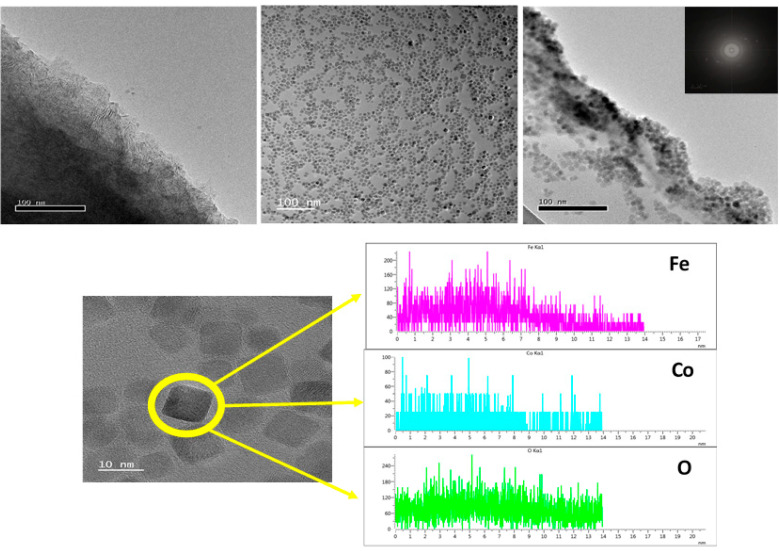
Transmission Electron Microscopy (TEM) images of the (**a**) WS_2_ sheets, (**b**) s-CoFe_2_O_4_@c-Fe_3_O_4_ NPs and (**c**) WS_2_/s-CoFe_2_O_4_@c-Fe_3_O_4_ nanocomposites shows the layered structure of WS_2_ and the size of the nanoparticles (NPs) is observed to be 10 nm, (**d**) the energy dispersive spectrometer (EDS) structural study of the s-CoFe_2_O_4_@c-Fe_3_O_4_ NPs. The inset of figure (**c**) showed its corresponding selected area electron diffraction (SAED) pattern. (**d**) The EDS structural study of the s-CoFe_2_O_4_@ c-Fe_3_O_4_ NPs confirmed the presence of the cobalt, iron and oxygen elements in the core-shell magnetic nanoparticles (MNPs).

TEM images of the WS_2_ sheets, s-CoFe_2_O_4_@c-Fe_3_O_4_ core-shell NPs, WS_2_/s-CoFe_2_O_4_@c-Fe_3_O_4_ nanocomposites are shown in Figure 1. As seen in Figure 1a, the WS_2_ sheets performed layered structures in micro size. The TEM images of core shell MNPs (Figure 1b) showed the cubic iron oxide shell. The size of the core shell NPs is evaluated to be ~10 nm. The spherical cobalt ferrites are expected to cover inside the core-shell structures. Here, the core-shell NPs were obtained by a seed mediation thermal decomposition routes, which was used as prepared CoFe_2_O_4_ NPs as template, then the cubic structured iron oxide NPs is grown as a shell. As seen in Figure 1b,d, the CoFe_2_O_4_ does not appear in the TEM mode. This could be considered as an obscured signal because of the thick shell. This assumption can be supported by energy dispersive spectrometer (EDS), which confirmed the present of the cobalt and iron and oxygen element in the core-shell NPs (Figure 1d). The TEM image of nanocomposites (Figure 1c) confirmed the s-CoFe_2_O_4_@c-Fe_3_O_4_ NPs attachments in the WS_2_ surfaces. In addition, its inset shows the selected area electron diffraction (SAED) pattern for the nanocomposites; lattice fringes corresponding to the WS_2_ sheets and core-shell NPs could be observed [44].

### 2.2. MTT Assay of WS_2_ Sheets and WS_2_/s-CoFe_2_O_4_@c-Fe_3_O_4_ Nanocomposites 

**Figure 2:**
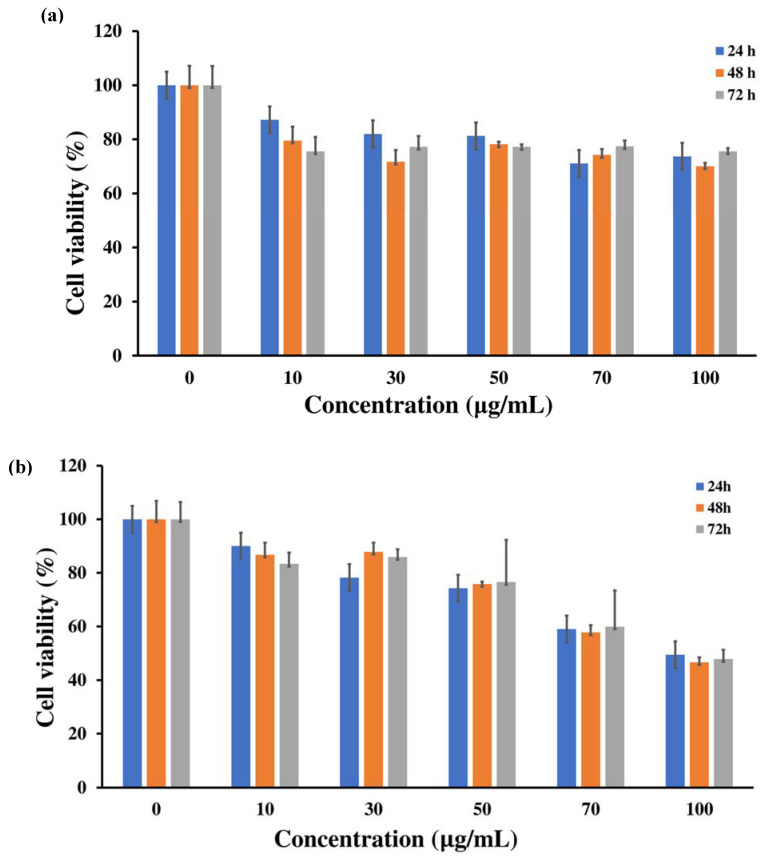
3-[4,5-dimethylthiazol-2yl]-2,5-diphenyl tetrazolium bromide (MTT) assay of the (**a**) WS_2_ sheets and (**b**) WS_2_/s-CoFe_2_O_4_@c-Fe_3_O_4_ nanocomposites on bovine cornea endothelial cells for duration of the 24, 48, and 72 h. The data were represented as mean ± SD (n = 3).

To study the cytotoxicity of the WS_2_ sheets and WS_2_/s-CoFe_2_O_4_@c-Fe_3_O_4_ nanocomposites, the bovine cornea endothelial cells were utilized. MTT assay of the BCECs exposed to the different concentrations of WS_2_ sheets and nanocomposites (range from 0 to 100 μg/mL) with treatment times of 24, 48, 72 h are presented in Figure 2. The result of cell viability of WS_2_ sheets on the BCECs (Figure 2a) shows low toxicity effects (~25% cell destruction) at the higher concentration of 100 μg/mL for 72 h [45]. Teo et al. [46] also reported previously that the cytotoxicity of TMDs (MoS_2_, WS_2_, and WSe_2_) was even lower than of graphene derivatives [47,48]. In their experiments, the MTT assays are evaluated on human lung carcinoma epithelial cells (A549) for 24 h of exposure, for which WS_2_ sheets show higher cell viability of 90.6%. The cytotoxicity of the WS_2_/s-CoFe_2_O_4_@c-Fe_3_O_4_ nanocomposites toward BCECs was shown in Figure 2b. As seen in the figure, BCECs cells exhibit different toxicity responses in different time and dosage of nanocomposites. The MTT assay result of nanocomposites revealed that toxicity is enhanced drastically in 100 μg/mL compared with the WS_2_ sheets. Moreover, cell destruction of ~50% was detected in 72 h of incubations. These results can be supported by research done by Yang et al. [43] on WS_2_@Fe_3_O_4_ nanocomposite coated with the mesoporous silica which showed no toxicity to the three types of tested cells (i.e., 4T1 murine breast cancer cells, HeLa human cervical cancer cells and 293T human embryonic kidney cells) even in 200 μg/mL. However, WS_2_ sheets and iron oxide nanoparticles are well known to be biocompatible materials and used often for in vivo cancer therapy. Then the increases of the cell death in nanocomposites could correspond to the trace amount of the cobalt content in the cobalt ferrites NPs [49,50]. In contrast, the WS_2_ was used as a biocompatible 2D material here for offsetting the toxic nature of cobalt add-up in the WS_2_/s-CoFe_2_O_4_@c-Fe_3_O_4_ nanocomposites [51].

### 2.3. WS_2_/s-CoFe_2_O_4_@c-Fe_3_O_4_ Nanocomposites Effect on BCECs Migration Capacity

**Figure 3:**
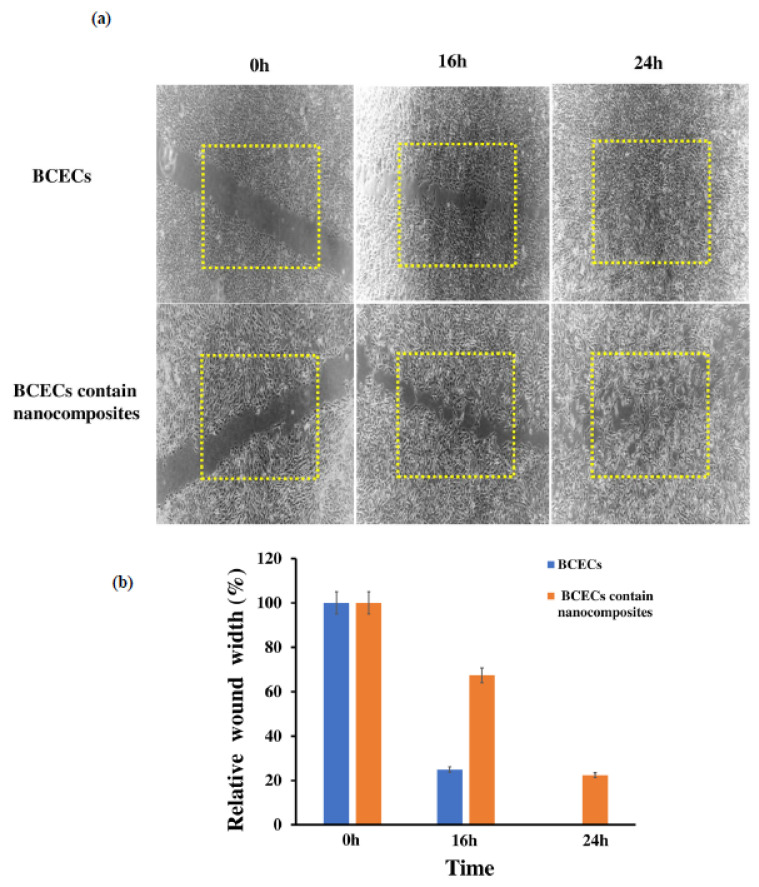
(**a**) The cell migration scratching assay on bovine cornea endothelial cells (**b**) quantitative analysis of BCECs migration represent the relative wound width calculated in relation to initial time (0 h). Results are expressed as mean ± SD repeated n = 3 experiments.

The cell migration response of the cornea endothelial cells is important for wound healing in eye surgery, ocular diseases, and angiogenesis [52,53]. The cell migration assay tested on BCECs was exposed with WS_2_/s-CoFe_2_O_4_@c-Fe_3_O_4_ nanocomposites for 24 h. The scratch wound assay of the layers of BCECs used as control, and the cell closures were scanned by optical microscope. The presented photograph was procured at 0, 16 h, 24 h after the wound formation in the culture of exposed BCECs and control (Figure 3a). The nanocomposites-treated BCECs show moderate wound closures compared to the control of ~33% closure and ~80% after 16 h and 24 h of incubation, respectively. Our results could be supported by previous studies on investigation the effect of nanoceria conjugated with heparin that suppressed the migrations of cornea endothelial cells [54]. The representative photograph of the BCECs used as control and cells contain nanocomposites shows that the BCECs loaded the gap by their migration and not by stretching the cell via increasing the cell size. Furthermore, the result shows that the WS_2_/s-CoFe_2_O_4_@c-Fe_3_O_4_ nanocomposites-treated BCECs attenuate the migration in transwell migration assay [55].

### 2.4. Photothermal Effect of WS_2_ Sheets and WS_2_/s-CoFe_2_O_4_@c-Fe_3_O_4_ Nanocomposites

**Figure 4:**
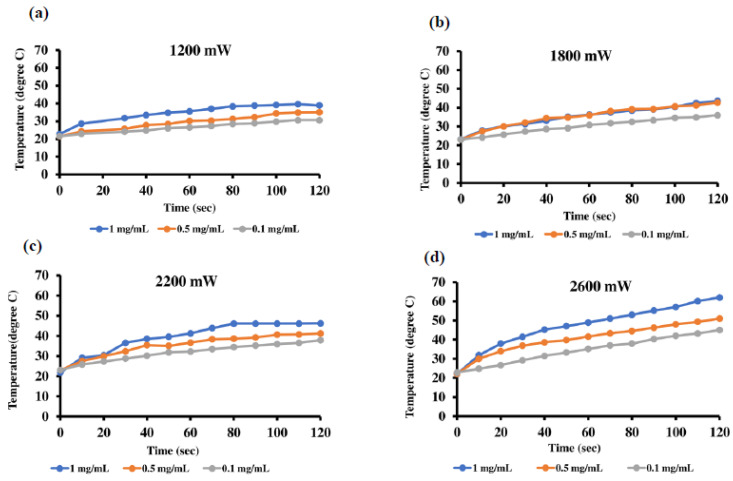
The photothermal evaluations of laser absorption of WS_2_ sheets in various laser power densities of (**a**) 1200, (**b**) 1800, (**c**) 2200 and (**d**) 2600 mW at concentration 1, 0.5 and 0.1 mg/mL.

**Figure 5:**
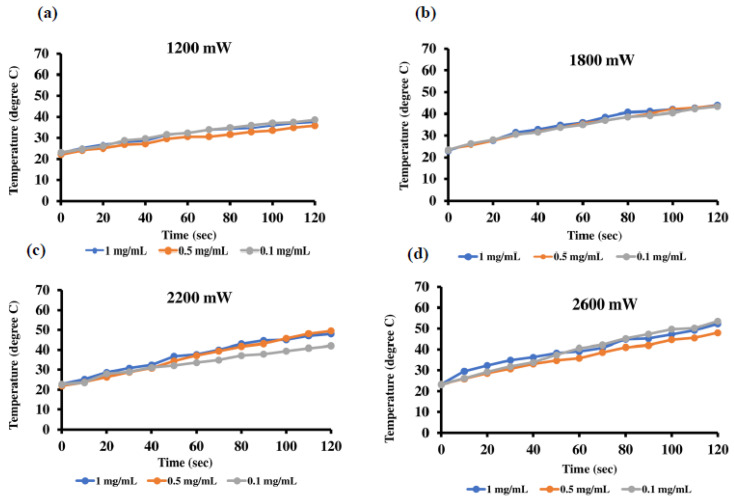
The photothermal evaluations of laser absorption of WS_2_/CoFe_2_O_4_ @Fe_3_O_4_ nanocomposites in various laser power densities of (**a**) 1200, (**b**) 1800, (**c**) 2200 and (**d**) 2600 mW at concentration 1, 0.5, and 0.1 mg/mL.

To investigate the photothermal response of the WS_2_ sheets and WS_2_/s-CoFe_2_O_4_@c-Fe_3_O_4_ nanocomposites, their NIR absorption was evaluated using 808 nm laser. The laser ablation evaluations of three different concentrations of the WS_2_ and WS_2_/CoFe_2_O_4_ @Fe_3_O_4_ nanocomposites (1, 0.5 and 0.1 mg/mL) for four different laser power densities of 1200, 1800, 2200 and 2600 mW were shown in Figure 4 and Figure 5, respectively. The results show an increasing trend of temperature via time in each laser when densities for all nanocomposites suspensions are applied. In other words, for concentration of 1 mg/mL of the WS_2_ sheets and WS_2_/CoFe_2_O_4_ @Fe_3_O_4_ nanocomposites suspension in DI water, in the same power density of 1200 mW at 2 min of laser irradiation, the temperature was found to be 17.4 °C and 15.6 °C, respectively. The increasing of the temperature rate is proved to be dependent on samples concentration, laser irradiation time, and power density. As reported previously, WS_2_ sheets and their composites exhibited an absorption in NIR regions (750–850 nm) which was higher than that for graphene in the same region [56,57].

The reason for the decreases of temperature rate in nanocomposites compared with the WS_2_ sheets could be due to the partial coverage of the WS_2_ sheet surfaces with the core-shell NPs. Also, when the magnetic core-shell NPs are attached electrostatically on the WS_2_ sheet surfaces, with proper distance between them, it avoids their aggregations via dipole-dipole interactions, which is a great benefit to manage the appropriate distance between the WS_2_ sheets for better NIR absorption when the WS_2_ sheets are multilayered. The experiments were repeated for three cycles under laser irradiation for each sample to confirm the equal temperature rate. Thermal stability of the samples shows no significant reduction in optical absorbance in each cycle and the temperature of the water under laser irradiation of reported power densities in two minutes shows elevation below 1 °C.

### 2.5. MRI Study

**Figure 6:**
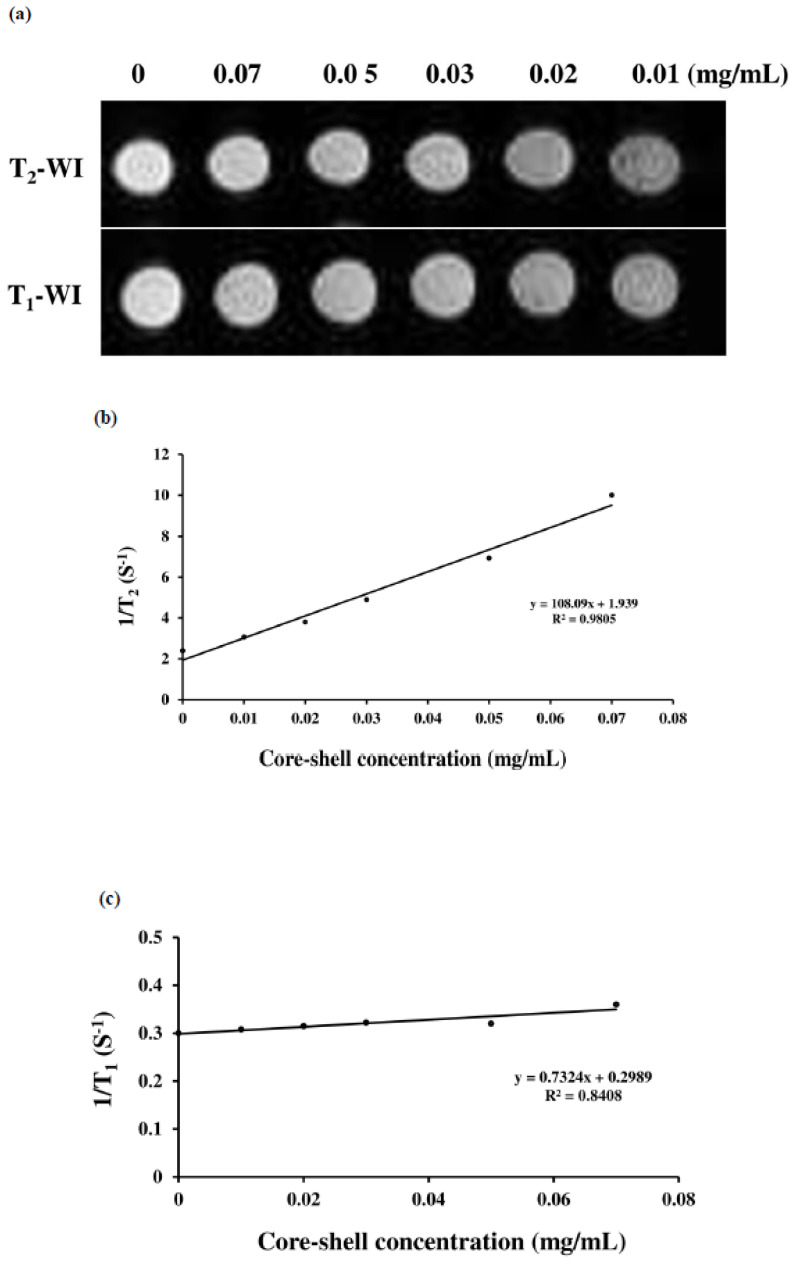
(**a**) T_1_ and T_2_-weighted MR images at five different concentrations of nanocomposites. (**b**) The linear fitting of relaxation rates (1/T_2_) of versus nanocomposites concentrations. (**c**) The linear fitting of relaxation rates (1/T_1_) of versus nanocomposites concentrations. The relaxivity values of r_2_ and r_1_ were obtained from the slopes.

MRI is a strong medical imaging technique that can provide great anatomical detail. It works by interaction of protons with the tissue’s surrounding molecules [58,59]. To improve the resolution and specificity of MRI, the use of the effective contrast agents is important. Recently, research attentions have been devoted to the design and synthesis of novel MNPs with higher saturation magnetization values that have better MRI contrast agents and shorten T_2_ relaxation times. However, superparamagnetic iron oxide NPs (SPIONs) have been used in clinics as negative contrast agents. Harrison et al. injected SPIONs coated polymers and tags with the fluorophore in the animal optical nerve for in vivo MR imaging [60]. Here, the nanocomposites of 2D/iron-based contrast agents in the core-shell form are designed to use as in vitro MRI contrast agents of the ocular system. Figure 6 shows the relaxivity measurements of WS_2_/s-CoFe_2_O_4_@c-Fe_3_O_4_ nanocomposites for various concentrations. The T_1_-weighted and T_2_-weighted images show concentration-dependent contrast. The results show which nanocomposites could act as a T_2_-weighted contrast agent. Figure 6b shows the 1/T_1_and 1/T_2_ relaxation diagram versus the nanocomposite’s concentrations. The longitudinal and transverse nuclear relaxivities generated from the slopes of 1/T_1_ and 1/T_2_ plots are approximately *r*_1_ = 0.73 mM^−1^ S^−1^ (R^2^ = 0.84), and *r*_2_ = 108 mM^−1^ S^−1^ (R^2^ = 0.98). The high *r*_2_ revealed the darkening effect of nanocomposites via different concentrations. The results show that the nanocomposites have the potential to be used as T_2_ MRI contrast agents in a diagnostic probe.

## 3. Materials and Methods

### 3.1. Materials

Iron acetylacetonate (Fe(acac)_3_), cobalt acetylacetonate (Co(acac)_2_), phenyl ether, benzyl ether, oleic acid (OA), and absolute ethanol were obtained from (ACROS ORGANIC, Morris Plains, NJ, USA), Tungsten hexachloride (WCl_6_) and 1-octadecene were purchased from (Alfa Aesar Inc., Haverhill, MA, USA), Oleylamine (OAm), hexane, ethylene glycol, 3-(3,4-dihydroxy)hydrocinnamic acid (DHCA), tetrahydrofuran (THF), NaOH, and phosphate buffered saline (PBS, concentration of PO_4_^2−^ ions (0.0067 M) were acquired from Tokyo Chemical Industry (TCI Co, Tokyo, Japan), (Fisher chemicals, Pittsburgh, PA, USA), (J.T.Baker, Phillipsburg, NJ, USA), (Sigma Aldrich, St. Louis, MO, USA) (ECHO CHEMICAL CO., LTD., Taipei, Taiwan), (SHOWA Chemical, Tokyo, Japan), and HyClone^TM^, Marlborough, MA, USA) respectively. The above chemicals were used without further purification. The 3-[4,5-dimethylthiazol-2yl]-2,5-diphenyl tetrazolium bromide (MTT) and Dulbecco’s Phosphate Buffered Saline (DPBS) were purchased from (Sigma Aldrich, St. Louis, MO, USA).

### 3.2. Synthesis of the Core-Shell NPs

#### 3.2.1. Cobalt Ferrite (s-CoFe_2_O_4_) Seed Nanoparticle Preparation

A quantity of 2.655 g Fe(acac)_3_ and 1.26 g Co(acac)_2_ were mixed in the presence of 60 mL phenyl ether, followed by adding 60 mL OAm. The mixed solution was added into the nitrogen-injected three-necked round-bottom flask and heated at 100 °C for 1 h, in order to remove the moisture. After 1 h, the solution was heated to the temperature 260 °C at rate 3 °C/min and heated to reflux at 260 °C for 2 h. Then, the solution was cooled down by the removal of the heat resource to the room temperature. The as-synthesized mixture was washed by ethanol and collected by the magnetic decantation twice for each sample to remove the residues of the unreacted chemicals. The black precipitation was redispersed in 30 mL hexane. The obtained product was labeled as spherical OAm-CoFe_2_O_4_.

#### 3.2.2. s-Cobalt Ferrite@c-iron Oxide (s-CoFe_2_O_4_@c-Fe_3_O_4_) Core-Shell Preparation

A quantity of 1 g Fe(acac)_3_, 3 mL seed solution (12.6 mg CoFe_2_O_4_), 20 mL OA, 20 mL OAm, 20 mL benzyl ether, and 1.75 mL ethylene glycol were mixed uniformly, followed by being added to the three-necked round-bottom flask, which was preheated at 100 °C under nitrogen atmosphere. After preheating for 1 h, the solution was heated to the temperature 290 °C at rate 6 °C/min and refluxing for 30 min. Due to the presence of ethylene glycol as mentioned above, as the temperature reached 240 °C, a vigorous evaporation would be expected. Thus, the nitrogen flow rate was adjusted to increase the flow rate to remove the vaporous ethylene glycol from flask. After refluxing, the solution was cooled down to room temperature and further washed with the absolute ethanol and magnetic decantation twice for each. The obtained powders were suspended in 10 mL hexane for storage, and labeled as cubic OA-CoFe_2_O_4_@Fe_3_O_4_.

#### 3.2.3. Ligand Exchange of Core-Shell Nanoparticles

A volume of 2 mL cubic OA-CoFe_2_O_4_@Fe_3_O_4_ (solvent: hexane) was added into a flask, which contained 3-(3,4-dihydroxy) hydrocinnamic acid/tetrahydrofuran solution (DHCA/THF, 0.125 g/15 mL). The solution was stirred with magnetic stirrer at 60 ℃ for 4 h. After the ligand exchange finished, the products were washed with ethanol containing a small amount of NaOH twice. The precipitation was further redispersed in 2 mL DI water. The samples were labeled as cubic DHCA-CoFe_2_O_4_@Fe_3_O_4_ [61].

#### 3.2.4. Preparation of WS_2_/Core-Shell Nanocomposites

##### WS_2_ Preparation

A quantity of 0.3966 g WCl_6_ was mixed with 20 mL OAm and 10 mL 1-octadecene at room temperature [45]. After 1 h of preheating and nitrogen injection at 100 °C, the mixture was heated to 300 °C and reflux for 30 min, followed by adding 0.4 M of sulfur/OAm solution. The reaction at 300 °C was further carried out for 1 h to form WS_2_ sheets. The solution was cooled down and washed by conducting the addition of absolute ethanol and centrifugation at rate 4000 rpm twice. The resultant solution was dried and annealed at 500 °C in the oven under nitrogen atmosphere for 2 h. Finally, the WS_2_ sheets could be collected to use in further work.

##### WS_2_/Core-Shell Nanocomposite Preparation

**Scheme 1:**
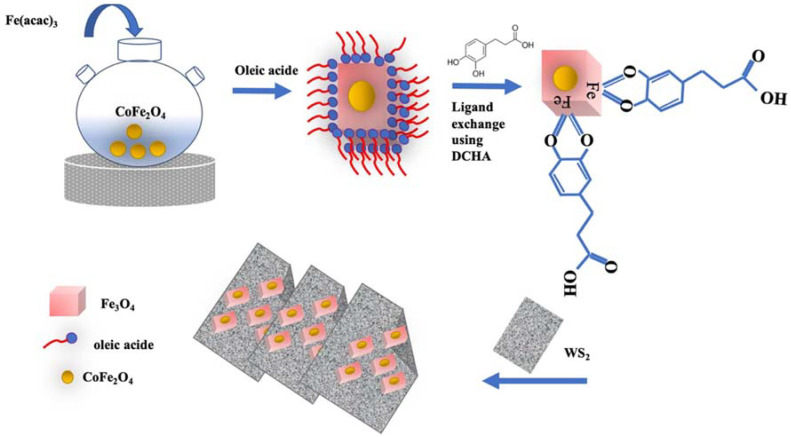
Shows the synthesis process of WS_2_/s-CoFe_2_O_4_@c-Fe_3_O_4_ nanocomposites.

To decorate the cubic DHCA-CoFe_2_O_4_@Fe_3_O_4_ between the layers of WS_2_ sheets, the few-layer WS_2_ sheets were sonicated in a bath-type sonicator before capping the cubic core/shell structured MNPs. The as-made few-layer WS_2_ sheets with phosphate buffered saline (PBS) (1 mg mL^−1^) were added to cubic DHCA-CoFe_2_O_4_@Fe_3_O_4_ in the ratio of 1:5 in weight. The mixture was stirred at room temperature for 24 h. The products were collected by magnetic decantation. The nanocomposites were labeled as WS_2_/spherical CoFe_2_O_4_@cubicFe_3_O_4_ (see Scheme 1).

### 3.3. Characterization Techniques

#### 3.3.1. Transmission Electron Microscopy (TEM)

The morphology, selected area electron diffraction (SAED) and energy dispersive spectrometer (EDS) nanocomposites was obtained with the TEM (operating accelerating voltage: 200 kV, Philips field-emission, Tecnai F20, electron gun of ZrO/W(100) Schottky type, resolution ≤0.23 nm (Philips/FEI Corporation, Eindhoven, The Netherlands). The aqueous samples suspended in DI-water were dropped on the carbon-coated copper grids (200 mesh), followed by dried at 80 °C for a few hours before entering the TEM chamber.

#### 3.3.2. Derivation of Bovine Cornea Endothelial Cells (BCECs)

The bovine eyes were collected from butcher (Taipei, Taiwan) and cleaned and fumigated by iodine solution. Then the eyes were washed with Dulbecco’s Phosphate Buffered Saline (DPBS) and the cornea were detached from the eyes followed by removal of the Descemet membranes. The 10 mL of trypsin were added to the membranes and incubated at 37 °C with 5% of CO_2_ flow for 45 min. The BCECs were collected using centrifuge for five minutes of 1000 rpm and dispersed in the 6 cm culture dish. The cells grew and increased during a two-week incubation.

#### 3.3.3. Cytotoxicity Measurements

The cytotoxicity of the WS_2_ and the WS_2_/s-CoFe_2_O_4_@c-Fe_3_O_4_ nanocomposites on the BCECs were assessed using 3-[4,5-dimethylthiazol-2yl]-2,5-diphenyl tetrazolium bromide (MTT) assay. The cells were cultured in the 96 well plates (10^4^ cells/well) and incubated in 37 °C with 5% of Co_2_ for 24 h.

Then the various concentrations of the WS_2_ and nanocomposites (10, 30, 50, 70, 100 µg/mL) were dispersed in the PBS and added to the cells. The MTT assay was done on BCECs incubated with the samples after 24, 48 and 72 h. Finally, the 10 µL of the MTT solution was added to each well and incubated under 5% CO_2_ at 37 °C for 4 h. The cells were washed by PBS followed by adding the dimethyl sulfoxide (DMSO) in each plate and shake for 10 min. The optical density was collected by inserting the plates in microplate reader (Bio-Rad S/N 21648, Pleasanton, CA, USA) with excitation wave length of 595 nm. [62]. Data were presented as mean standard deviation (±SD) of three experiments. The following equation was used to calculate the survival BCECs from the absorbance collected by microplate:

MTT assay(cell viability%)=(sample abs 595 nm)/(control abs 595 nm) × 100

#### 3.3.4. BCECs Migration Assay

In vitro BCECs scratch assay or migration assay was performed in 6 cm round culture plates. The procedure was started with the seeding of the 10^6^ BCECs in each dish and incubated for 24 h. Then 100 μL of the WS_2_/s-CoFe_2_O_4_@c-Fe_3_O_4_ nanocomposites was dispersed in PBS and added to the culture dish. The cells and nanocomposites were incubated for 24 h until the nanocomposites were taken up by the BCECs. Straight lines were scratched in the cultured dishes (control and dish with the nanocomposites) by a sterile pipet tip of 100 μL. The cell migrations were targeted for 24 h. The collected images of the scratches were achieved at 100× magnification followed by further analysis by Image J software. Each experiment was repeated in triplicate.

#### 3.3.5. Magnetic Resonance Imaging of Nanocomposites

To achieve the WS_2_/s-CoFe_2_O_4_@c-Fe_3_O_4_ nanocomposites MRI relaxivity, a 7 Tesla (Bruker BioS pec 70/30 US, Billerica, MA, USA) scanner was used. The phantoms are prepared by the various concentration of the nanocomposites (0.07, 0.05, 0.03, 0.02, 0.01 mg/mL) dispersed in deionized water (DW) by adding 1% of agarose gel in the 0.5 mL plastic container. The relaxation times of hydrogen protons in the aqueous solution (T_1_ and T_2_-weighted) were measured at repetition time TR: 4000 ms; TE: 18 ms. To calculate the data, the obtained T_1_ and T_2_ maps were analysed presuming a mono-exponential signal decay. The maps were recognized using six-spin echo (SE) images of TE and TR. The T1 and T_2_-weighted images were analyzed using a non-linear least-square curve using pixel intensities basis (Levenberg–Marquardt fit) using MATLAB (MathWorks inc. Natick, MA, USA) [59]. The r_1_ and r_2_ relaxivities were calculated from the slop of 1/T_1_ and 1/T_2_ (i.e., reciprocal for T_1_ and T_2_ relaxation times) versus the nano composite’s concentrations. The images were acquired by designing a sequence of slice thickness of 1 mm and a matrix size of 128 × 128 over FOV of 6 × 6 cm^2^.

#### 3.3.6. Near Infrared (NIR) Experiment

The WS_2_ sheets and WS_2_/s-CoFe_2_O_4_@c-Fe_3_O_4_ nanocomposites with concentrations of 0.1, 0.5, 1 mg/mL were dispersed in 1 mL DW and ultrasonicated until complete dispersion in the solution was achieved. The suspensions were irradiated by 808 nm NIR laser system (Arno Electro-Optics Ltd. Taipei, Taiwan) under four various power densities of 1200, 1800, 2200, 2600 mW. The temperature of the samples’ suspension in the DW were monitored using k-type thermocouple (TM-947SD) (Lutron, Taipei, Taiwan).

## 4. Conclusions

The WS_2_/s-CoFe_2_O_4_@c-Fe_3_O_4_ nanocomposites were synthesized via chemical routes. The TEM showed that the core-shell NPs with the size of the 10 nm were pinned up on the WS_2_ sheet surfaces. The MTT cell viability assay of the nanocomposites on the BCECs showed the 50% cell viability in 100 µg/mL at 72 h of incubation. The cell migration studies of the nanocomposites exposed to the BCECs showed wound closure of 80% compared to the control. The photothermal studies of the WS_2_/s-CoFe_2_O_4_@c-Fe_3_O_4_ nanocomposites showed temperature elevated trend in each power density and the temperature rates were maximized for the lower concentration of 0.1 mg/mL. In the MRI of WS_2_/s-CoFe_2_O_4_@c-Fe_3_O_4_ nanocomposites the r_2_ relaxivity value was calculated to be 108 mM^−1^ S^−1^. The results showed that nanocomposites have the potential for use in laser treatments and as a T_2_-weighted MRI contrast agent for ocular systems.
